# A 3D Model of the Jaw Applied to Paediatric Dentistry

**DOI:** 10.3390/bioengineering9040143

**Published:** 2022-03-28

**Authors:** Begoña Gómez Legorburu, Alberto Adanero Velasco, José Ramón Mérida Velasco, Paloma Planells del Pozo

**Affiliations:** 1 Independent Researcher, 28039 Madrid, Spain; beguss@hotmail.com; 2 Clinical Dentistry Department, Biomedical and Sciences Faculty, European University of Madrid, 28670 Madrid, Spain; 3 Department of Anatomy and Embriology, Faculty of Medicine, Complutense University of Madrid, 28040 Madrid, Spain; mvlopera@med.ucm.es; 4 Clinical Specialties Department, Faculty of Dentistry, Complutense University of Madrid, 28040 Madrid, Spain; pplanells@odon.ucm.es

**Keywords:** 3D model, jaw, paediatric dentistry, virtual reality, anatomy

## Abstract

As education and knowledge are adapted to new education systems, as per the Bologna Plan, new technologies are required for educational support. In dentistry, the creation of virtual simulators can advance understanding in areas like anatomy. With this aim, a three-dimensional virtual model of the maxilo-mandibular system was created, based on a real infantile specimen. Once this model was developed, we applied this virtual structure to a teaching tool in a dentistry subject.The main objective of this project is the creation of a virtual model of the jaw, based on a real and infantile subject that serves as an educative tool in the scope of Paediatric Dentistry Anatomy. The secondary aims were to evaluate this model as an educational tool in a paediatric dentistry subject. For the main objective we obtained tomographic cuts of the craniofacial skeleton of a three-year-old girl that were transcribed to the program Amira 5.3.0. On the cuts, we segmented and named all the mandibular structures: jaw, temporary teeth, and permanent teeth. For the secondary aims we developed virtual clinical cases based on this mandibular model and gave a questionnaire to 29 dentistry students in order to evaluate the tool. A total of 512 cuts were obtained in sagittal and coronal planes and 309 in the cross-sectional plane of a thickness of 0.625 mm. In different colours, we segmented the total 25 structures to generate a three-dimensional mandibular model. For the questionnaire, the results of the students’ satisfaction of the tool were high, with an overall score of 8.5 out of 10. The educative system based on the Bologna Plan is a reality. The self-training based on test and error, is a strategy of extreme utility for the student. With an interactive model, the student is able to value his knowledge instantaneously, and the presence of a professor is not essential at all times. Through this real model, we have described the anatomical study of temporary teething, as well as its interactions with the developing permanent dentition, in a three-dimensional form. The students’ satisfaction of the teaching tool was high.

## 1. Introduction

It is well known that, nowadays, informatics is present in all areas. We are not able to conceive a university campus without its computer science area, or a teacher without its technical support. With the arrival of this digital age, obtaining digital data based of the human body becomes essential.

Despite this, the digitalization of the human body is quite complex [1]. Given this necessity, the concept arises from “Virtual Humans”, like a digital interactive model that integrates anatomical, physiological and biomechanics characteristics of the human body. Thus, we can find: The “Visible Human”, “Physical Human” and “Physiological Human”. The first generation allows the visualization of anatomical structures in three dimensions, whereas the other two try to reproduce the physical and physiological characteristics of the human body, needing a more complex technology [2].

The development of the “Visible Human Project” (VHP) at the end of 80 years, on the National Library of Medicine of the United States, has allowed us to obtain a gallery of digital images coming from cadavers (a man and a woman), which includes digitalized images of cryosections, transvers computer tomographies (CT), and images of magnetic resonance [3,4,5]. This project was the departure point of a new concept, such as virtual education in the field of the anatomy, that offers considerable advantages to conventional education based on cadavers.

In addition, the three-dimensional reconstructions are very precise, enabling the student to visualize any organ within all the planes of the space and their relationships. For that reason, by dissemination through the database created by the Center of Sciences of the Health at the University of Colorado, in December of 1995, the VHP is being used by students, professors, industrial groups in more than a hundred countries [1,2,4,5].

In 2000, the University of Ajou in Korea and the Korean Institute of Sciences and Technology of the Information began the project “Visible Korean Human” (VHK), which ended in 2005, from a cadaver of a 33-year-old patient [1,6,7,8]. It is known that China has the largest population in the world; for that reason, in 2001 in the 174th Scientific Conference of Xiangshan, the idea was conceived to create a “Visible Chinese Human”, which would suppose the creation of a digital base of images of a representative Asian man and woman. The image of the adult man was concluded in 2002, and that of the woman in 2003 [1,2,4,5].

In Germany, the University Medical Center of Hamburg Eppendorf created the “Voxel Man”, an anatomical data base with three-dimensional images of hi-resolution, with applications in surgical simulations and virtual corporal models from the images of the man of the VHP [2,9,10].

In the context of projects from “Virtual Human” and the need for new education pedagogies adapted to the new technologies, we propose the creation of a three-dimensional model of a jaw of a patient in growth that serves as an educative instrument in the scope of anatomy, paediatric dentistry and operative dentistry.

The main objective of this project is the creation of a virtual model of the jaw, based on a real and infantile subject that serves as an educative tool in paediatric dentistry, in the field of anatomy. The secondary aims are to create an educational tool based on this mandibular model and evaluate a questionnaire answered by the students.

## 2. Materials and Methods

### 2.1. 3D Model

For the main objective, we made a three-dimensional reconstruction of the jaw, from the executed tomographic cuts from a head of a three-year-old girl.

For doing so, we needed the program Amira 5.3.0, (Thermofischer Scientific, Waltham, MA, USA), installed in a Toshiba laptop, model Satellite L675–11N of 64 bits, with an Intel processor Core I5 M480 to 2.7 GHz and with a Ram memory of 4.00 GB, with the operating system Windows 7 Home Premium.

Software Amira, installed and yielded by the company D.I. Abadía, allows us to visualize the images in the three planes of the space, coronal (xy), sagittal (yz) and cross-sectional (xz). In each plane, the user can interact, and move cut to cut to rigorously examine all the bony structures and cavities at cranial level. In addition, it allows a three-dimensional reconstruction of the image, transforming the bi-dimensional unit pixel, to a three-dimensional unit called voxel.

A voxel (volumetric pixel) is the cubical unit that composes a three-dimensional object. It constitutes the sortable minimum unit of a three-dimensional matrix and is, therefore, the equivalent of the pixel in an object 3D.

In order to create an image in three dimensions, for images in grayscale, voxels must undergo a transformation of density based on the different structures of the body. This information confers different values of density from each voxel. Like the pixels, voxels do not contain their position (x and z) in space, but this position is deduced by the position of the voxel within the data file.

The captured anatomical structures in the scanner must become delimitated and reconstructed in 3D areas. This process is called segmentation. In our project we have segmented the jaw. All these areas were delimited in two planes, manually, and generated semi automatically to a three-dimensional system. They were catalogued with a name, for example: jaw, or the name of each tooth and germ.

The segmentation of images was defined as the partition of an image in constituent regions, which are homogenous with respect to some characteristic like an intensity or a texture.

The labelling allowed us to obtain the classification of pixels, which is a desirable objective in the treatment of medical images since, normally, the classification becomes necessary for disconnected regions that belong to the same tissue.

We have segmented the mandibular bone, with all the temporary teeth, as well as the germs of the definitive teeth in the formation. For ithist, an arbitrary code of colours has been used, as well as the anatomical denomination of the different structures.

For the secondary aim, we developed an educational tool for the students in the fourth year of a dentistry degree and made a questionnaire in order to evaluate the student opinion of the 3D model.

### 2.2. Drawing Up of the Didactic-Practical Innovation Units

Contoured drawings of the different tomographic slices of the upper and lower jaw were designed in order to help students visualize and become familiar with each diseased area. Medical histories with complementary diagnostic procedures and CT scans of paediatric patients were reviewed in order to select the clinical cases.

**Figure 1:**
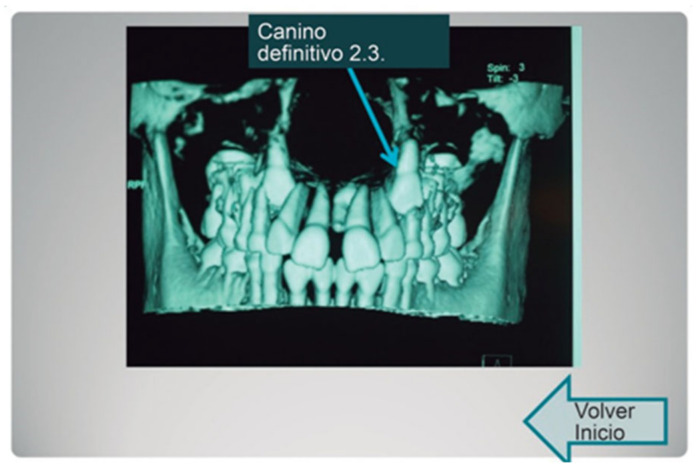
Example of the CT image chosen for the clinical cases.

The cases presented were selected because they were the most demonstrative for learning about, and defining, the anatomical structures in the area of the mouth of the growing patient (Figure 1).

**Figure 2:**
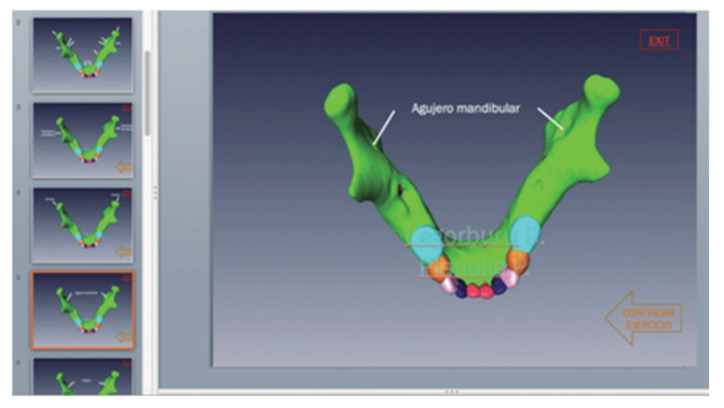
Example of 3D image chosen from one of the interactive presentations.

In parallel, using the tridimensional images of the jaw, we designed the interactive templates with hyperlinks, so that the student could navigate and choose an option for each assumed clinical scenario, in order to define a more precise diagnosis as a result of identifying anatomical structures (Figure 2).

**Figure 3:**
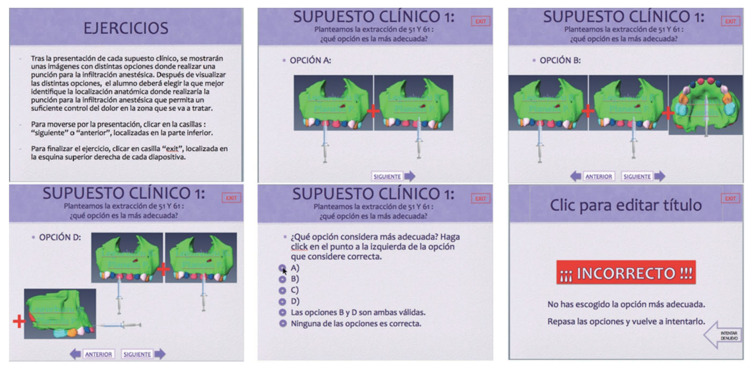
Simulated sequence taken from one of the interactive presentations.

With regard to the methodology used for the objective proposed in this research, and based on the choice of tridimensional images of the jaw previously mentioned, a presentation was designed with hyperlinks in which different clinical situations were envisaged. In this assumed clinical scenario, the student was questioned on the anatomic location for the puncture with a view to achieving effective anaesthesia, which would allow the proposed treatment to be carried out. The student was able to choose the answer from various options and, using the hyperlinks, find out if the answer was correct or not (Figure 3).

After the development of the tool, there were a questionnaire for the students. It was answered after the clinical cases were observed by the students. A total of 29 students answered the survey.

A descriptive analysis was made, with the questions of the survey in order to improve the educational tool for the students. An Excel sheet with all the answers was used to calculate the means and the total results of the questionnaire.

## 3. Results

### 3.1. 3D Model

From the tomographic images obtained and transferred to Amira 5.3.0, we collected all the data of the cranial skeleton divided in cuts: coronal (xy), sagittal (yz) and cross-sectional (xz), of great utility to work in the segmentation and the labelling.

A total of 512 cuts were obtained in coronal plane (xy) and sagittal (yz) and 309 in the cross-sectional one (xz).

Also, the program allows us to work at a three-dimensional level or a bi-dimensional level, in its different screens.

**Figure 4:**
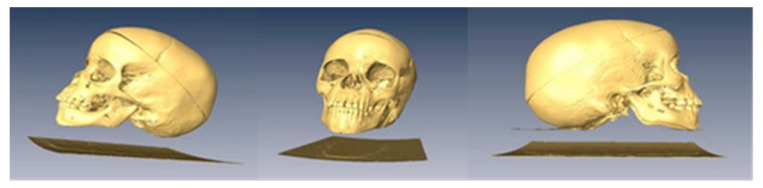
3D reproduction of the cranial skeleton of the cadaver in lateral and front views.

Also, the following figure shows the skeletal structure of that we started with, which is a three-dimensional reconstruction with one of the tools of the program (Figure 4).

From the beginning screen, we accede to our segmented screen, from where we will work to delimit our mandibular structure and the dental organs. Once in this screen, we firstly segmented the mandibular bone and then the permanent teeth and germs of temporary teeth.

In our study, we began with the sagittal cut, because we thought that in this cut, the different structures are appraised with greater clarity. In order to do so, we avoided the rest of the cuts, and, in the screen, we placed only the axial one. Then, we extended the image for a greater visualization at the time of beginning the segmentation. With the cursor of the mouse, we drew and delimited structures.

As we can see, there exists a red area, that we are delimiting and to which we will assign name and colour (a “label”), that is reflected in the left superior part of the screen (blue box of the figure). The allocation of name and colour is arbitrary. In our work, a total of 25 structures have been segmented. They include the mandibular bone, the totality of the inferior temporary teeth, as well as all the corresponding germs of the definitive teeth in their respective evolutionary stages.

**Figure 5:**
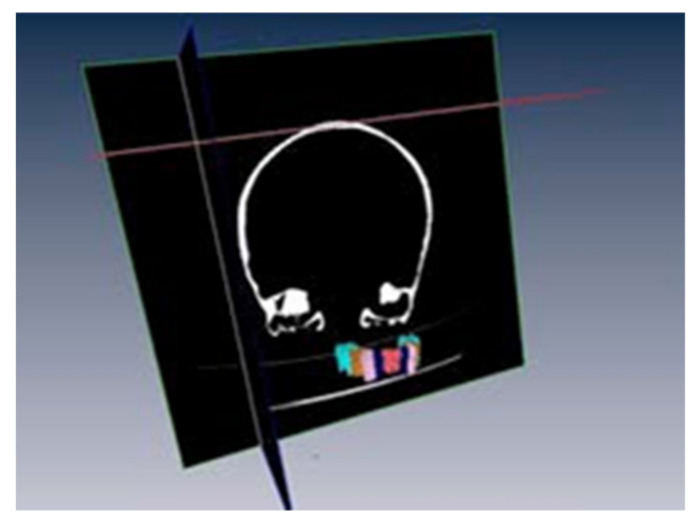
Temporary teeth.

**Figure 6:**
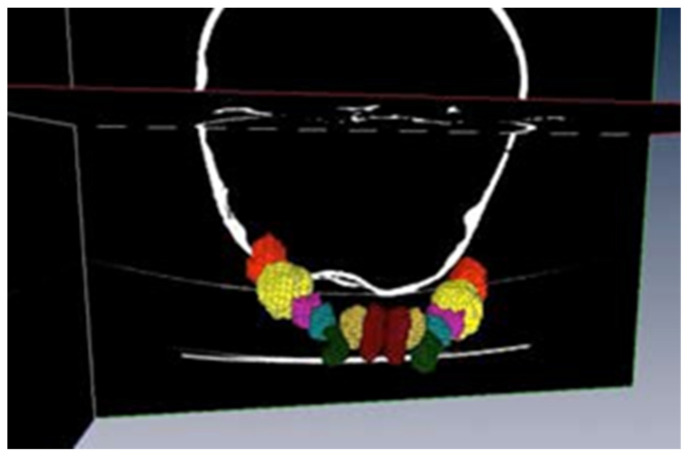
Germs of permanent teeth.

**Figure 7:**
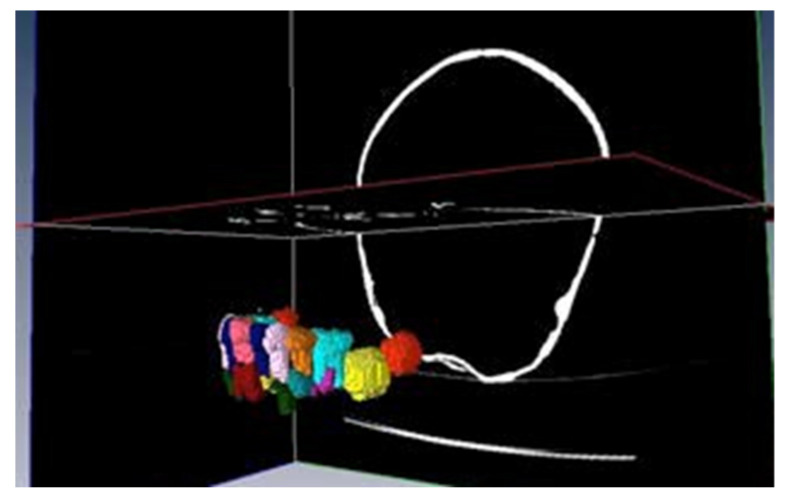
Temporary teeth and germs.

**Figure 8:**
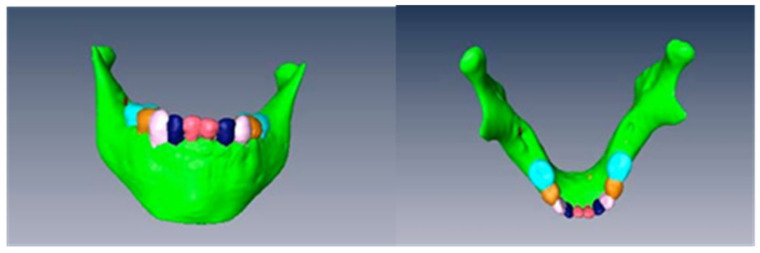
Frontal and coronal views of the final structure.

This work finally leads to the creation of a totally interactive three-dimensional structure of the jaw. The following figures demonstrate the evolution in the determination of each one of the elements until the final goal of the total mandibular structure (Figure 5: Temporary teeth, Figure 6: Germs of permanent teeth, Figure 7: Relationship between temporary teeth and permanent germs). Figure 8 shows the final result in a frontal and coronal vision.

### 3.2. Educational Tool

After adjusting the didactic material developed, the teachers proceeded to present the clinical cases to the paediatric dentistry students, using seminar sessions.

Once the seminars were given, the interactive presentations were uploaded onto the Virtual Campus so that the students could have the opportunity to self-evaluate their acquired knowledge and abilities.

A survey of the students was then carried out and their opinions were evaluated regarding the exercises, tools and the acquisition of skills, compared with traditional learning systems.

There were 29 students in the initial group of the study, and 100% of the students in the group answered the survey. There were three clinical cases, and three questions on each clinical case. All of the questions concerned the evaluation of the educational tool, and there was an open question about possible suggestions.

The most important answers and the results on the questionnaire are shown on the next tables and figures.

**Figure 9:**
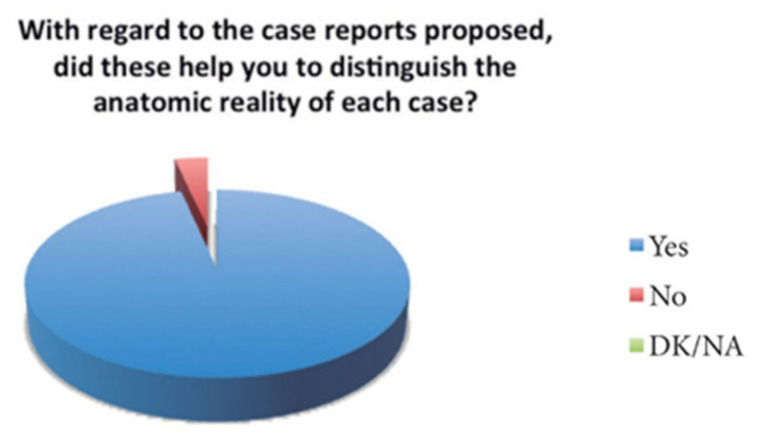
Representation of the percentage of responses.

**Figure 10:**
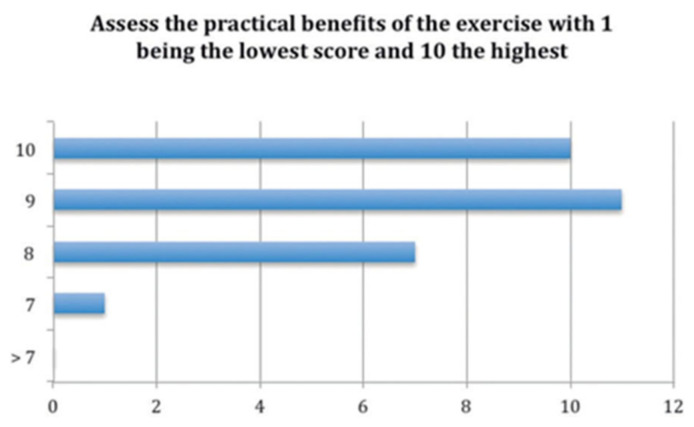
Representation of the students’ scores for the exercise (expressed in number of students).

Over the total number of the students, almost all of them (28 of 29) answered that this tool helped them to distinguish the anatomical structures (Figure 9), and the score of the clinical tool based on the 3D model was 8.5 out of 10 (Figure 10). The open answer question will be important in order to help us improve the tool for the students (Table 1).

## 4. Discussion

Learning Anatomy in a conventional way has been based on the use of atlas and dissections. An atlas provides clear and detailed images of all the elements of study, but with the limitation of the visualization in two dimensions. The dissections help the student to become familiar with reality, since they can verify the spacial organization of the study structures, their relationships, and insertion. However, the problems include temporality, and the difficulty of the obtaining of specimens [1,2].

In order to try to gear the student and the professional person towards a more dynamic learning adapted to new technologies, the idea arose to create anatomical data bases that allowed, by means of a connection in the network, us to accede to real specimens that have been previously digitized. Thus, in 1987 the NLM (National Library of Medicine) raised the necessity to create a virtual library of biomedical images. This one would be based on the creation of a data base of complete images of a man and a woman; it is what we know as the “Visible Human Project”. This project includes images taken from computerized tomographies, magnetic resonances, and photographs of cryosections of cadavers. In collaboration with the Medicine University of Colorado, in 1994, they acquired the body of a man and later one of a woman. With the intention to avoid a tissue deterioration, the body was put under treatment with formalin and anticoagulants, until arriving at the University where the work with him would begin. The magnetic resonance was done first, taking images in the axial and coronals planes, and the tomography cuts were made. Cross sections were taken every 3 mm for the head and neck, thorax, abdomen and pelvis, and every 5 mm in the inferior extremities. Later, the cadaver was congealed and tomography cuts were taken again. This time, the thickness of the cuts was 1 mm to match the previous acquired sections with the cryosections and that would take this thickness a year later [11]. Once the images of the different sources were obtained, they were processed digitally, obtaining a three-dimensional reconstruction, as well as the pertinent cuts. Then, a woman was created. The unique difference is that the sections were taken every 1/3 mm instead of 1 mm [12].

Years after the creation of the VHP, new models were generated with the idea of improving those that had already been created. In 2001, the University of Ajou in Korea, in collaboration with the Korean Institute of Science and Technology of Information, created Visible Korean Human (VKH), from the cadaver of a 33-year-old man who had died of leukemia [7,8]. With this project, they tried to improve the images obtained in the VHP, since, for example, the sections did not include ranks inferior to 0.33 mm, and the colours were not real due to the formalin injection for the maintenance of the corpse, and did not include segmented images [7]. For this reason, they did the magnetic resonance first, with cuts of 1 mm without leaving no a gap between each section, as happened with the tomographic cuts carried out each 1 mm. A preservative substance was added in the body. After freezing the cadaver, the cryosections were taken, this time every 0.2 mm. Once all the collected data were digitized, the researchers segmented important structures (skin, bones, liver, lungs, kidneys, arteries, urinary, respiratory, digestive system, brain, heart), which had not been realized in the VHP [7,8].

With the idea to create an anatomical and representative database from the Asian population, Chinese Visible Human (CVH) was created. This project includes a normal man and a woman, it started in 2002, with the creation of a man, and in 2003 the woman was created. Both were young, healthy specimens that were adjusted to what would be a real representative population. In contrast to the Korean project, the Chinese cadavers were treated with a red gelatine solution, to improve the visualization of the arterial and venous circuit. The sections of the resonance took place every 3 mm, and the tomographic ones every 1 mm. The cryosections were less precise than the Korean ones, because they had a thickness of 0.5 mm for head and neck, 0.1 mm for the base of the skull and 1 mm for the rest of the body, in the man and 0.25 mm for head and 0.5 mm for the rest of the body in the woman. Later, the data were digitized and reconstructed three-dimensionally, but without segmenting [1,2,13].

In our project, we have obtained the images only from tomographic cuts, from a skull that is in optimal conditions of conservation. In addition, it is important to emphasize that until now there is no similar model based on a real specimen of a subject in infantile age. 

In addition to the importance and innovation of the described works, as digital databases to world-wide disposition, an additional step was the use of the obtained images as a sustenance for real volumetric models that were used for the learning of the diagnostic and surgical skills [14].

A revolution in this area is the creation t the University of Hamburg-Eppendorf, Germany, the system “Voxel Man” [2,10]. The project arose prior to the existence of the VHP, due to the perseverance of Professor Karl Heinz Höhne who created a three-dimensional simulation of a brain of a live subject from tomography cuts. However, the revolution took place in the 1990s when the tomographic images and resonance of the VHP were obtained. From them they created systems of segmentation and visualization to build simulators and interactive navigators. They began by simulators of the brain and torso, in which surgeries could be planned, to cross venous, arterial systems, to work with endoscopy, to study the anatomo-physiology of the extremities with a great approach to reality. They created three simulators, with the concept “See and Feel the Difference”, of the average ear, another one of sinusal endoscopy, and a dental one, in which the user feels and works on three-dimensional reconstructions with the incorporation of hardware based on virtual reality and systems of detection of pressures, known as haptic systems [9].

From 2009, interactive dental models allowed an education of dental skills from a feedback system that allows a trustworthy form to feel the application of force on the tooth or bone. The differences between enamel, dentine, and carious tissues are appraised. In addition, it is equipped with a pedal, which enables adjustment of the speed of the instrument, and the creation of real situations, such as a decay or periapical surgeries [9,15].

Concerning simulation of the maxillofacial bone we found more examples in the literature that demonstrate that the three-dimensional models are a novel and quite useful tool. From the Virtual Chinese Human, a model of a jaw (similar to the one of our project) was created, but using, like the auxiliary program, Photoshop, and without making the segmentation of the structures [16]. With a similar methodology to ours, researchers at the University of Ontario constructed a model of a head and neck from a cadaver of an edentulous man, based on tomographic images. Using Amira, they segmented and labelled the different structures and they ended up with a three-dimensional reconstruction. They obtained 70 segmented structures, including soft structures (muscles, glands) that can be added and cleared for a correct anatomical study. This type of tools allows researchers to change sizes, to turn the models for one better visualization of the structures, to modify densities to see anatomical relations, to interact in the different planes, and to work in two and three dimensions [17].

Of interest at the anatomo-physiological level is the work of the University of Auckland, where researchers created a virtual model for the study of the masticatory system. For it, they generated a hypothetical bony model based on the anthropometric measurements of more than 1000 men of 17 ethnic groups of ages between 20 and 30 years. Once the digital bony structure was obtained, the dental pieces were derived from the scan of the coronary surfaces of a subject, whose occlusal faces were drawn up. Since the objective of this study was the evaluation of the temporomandibular system, mainly at the muscular and articular level, the authors emphasize that the precision in the one dental example is not a fundamental aspect. It is important to know that the muscular structures were taken from the archives of the Visible Human Project and that they fit with the aid of anatomists [18].

Virtual models can also be used for the learning of clinical skills. With the simulators and navigators of the Voxel Man, many surgeons have planned surgeries and endoscopies. Diverse studies compare the effectiveness of working on models based on cadavers or virtual models, obtaining very good results when working on the virtual system, since it allows better visualization, the reproducibility of the situations, and lower cost [16]. If we associated a haptic system with software or phantoms that allow the control of the tact and the exerted pressures, as well as to equip it with stereoscopic properties, we will have a similar situation to reality [19,20].

In dentistry, we have worked on phantoms with plastic dentures, which, although they approached reality, have some limitations. For this reason, virtual simulators were originated with the advantage that they allow the evaluation of the work carried out, by their visualization in the computer, and with the importance of feedback [21]. DentSim is a simulator created in the 1990s, that consists of a dummy with head and torso with Kavo dentures, a turbine and a hand piece Kavo, an adjustable tray, an air and water syringe, aspiration, lamp, and a computer to generate the virtual reality. While the student prepares the cavity on the dentures, software creates a virtual image of the preparation in the computer, besides enabling it to be visualized in 3D, and granting a qualification. In this way, the student can quickly evaluate his errors, improve them, and see his development [22]. Diverse studies have valued the effectiveness of this system like instrument of learning of skills. In 1998, the University of Pennsylvania began to use it with degree students and obtained satisfactory results [23]. A study realized in the University of Columbia demonstrated the effectiveness of DentSim when evaluating the progression in the learning time spent between a control group that realized the cavities on traditional phantoms, and a study group that worked on the simulator (spending less time). They attribute the advantage mainly in the immediate self-evaluation, since with the traditional method the students experience a delay until the professor makes the evaluation, therefore losing the possibility of carrying out more practice during this time [24].

A step forward supposes the incorporation of haptic systems to these virtual simulators. The haptic systems allow the operator and the computer to interchange mechanical energy. The majority of the systems are based on joysticks, thimbles or pens, which, depending on the time and the position in which they are, give information on the texture and surface in which they are working virtually. Traditionally, these systems have been used in surgeries. The University of Dentistry of Iowa in collaboration with the School of Engineers created a haptic system based on a computer connected to a dummy and joystick as a rotatory instrument with haptic properties. The users affirmed that the work sensation was quite realistic on different dental tissues, noticing the different textures and vibration; something that demonstrates that these systems could be very useful in education [25].

These types of systems have been extended to the different areas of dentistry, especially in implantology, periodontology, and endodontics [26,27,28,29]. Interesting work was carried out at the University of Tesalonica, where reesarchers created a virtual model of a head from the images of cryosections of the Visible Human Project, which could be modified based on the patient from two photographs taken of the subject. In addition, it allowed the researchers to model the teeth, to obtain different visions, and to choose the work bur, generating a real situation [30].

Konucseven et al. [31] in Turkey, created a complete virtual system with dental applications. The user can modify the rigidity, damping, coefficient of friction, and module of elasticity of different tissue, to be applied to the haptic system. The dental model was created based on a system of voxels. The visualization in all the planes, as well as any type of variation in the aperture angle of the jaw, increases and reductions, are tools incorporated into the software. In addition, instruments similar to those used in dentistry were designed virtually. After teaching a group of students how to use it, a questionnaire was given to them to evaluate practice with it. The majority of the users obtained a removal and detection of the decay in a satisfactory way; nevertheless, they found the system a little troublesome to familiarize themselves with, because of the use of the associated haptic system.

Using the Voxel Man, Pohlenz et al. created a model with periapical infections in certain teeth. Using haptic systems, a “ball-point pen” as a hand piece and a pedal to regulate the force, the users made the apicectomies of these teeth. At any moment the user could verify the direction of the bur, and the relations with the different structures. After giving them a survey to evaluate the satisfaction in their use, the majority of the participants reported that they found the system very useful [15]. We think that this system is of extreme importance since it is not based on finite systems or digital reconstructions; the images has been obtained from real tomographic cuts.

Until now, we have been able to verify that the studies based on virtual models suppose a tool to be very useful in the field of the anatomy and in the acquisition of manual skills. However, the found systems are based on adult specimens. For this reason, we think that our project would constitute an advance in these areas. The study of a developing jaw, the evolution in the eruptive sequence, and the formation of the dental germs are fundamental aspects in the learning of the anatomy of the maxillofacial system, as well as in Paediatric Dentistry.

As a future idea, we would like to segment and label the maxillary bone, to be able to create a complete model of the maxillofacial system. In addition, it would be interesting to generate a software of simulation similar to the one of the Voxel Man, in its dental version, that would be pioneering concerning an infantile subject, and an online library with easy access to the learning of anatomy and embryology related to Paediatric Dentistry. In addition, we believe that the use of Amira is an advance as far as interactivity is concerned, and not only a tool with which one can segment and label. With it, the student could handle the three planes of the space in an intuitive and simple form.

With the intention of improving clinical application, the future ideas of our group include creating a haptic system similar to the ones created in Germany, Turkey or Iowa, but working on an infantile model, which would constitute a pioneer in this field.

## 5. Conclusions

The educative system based on the Bologna Plan is a reality. The self-training based on test and error is a strategy of extreme utility for the student. With an interactive model, the student is able to value his knowledge instantaneously, and the presence of a professor is not essential at all times. Through this real model, we have described the anatomical study of temporary teething, as well as its interactions with the developing permanent teething, on a three-dimensional form. The images secured on the germs of the developing permanent teeth in this investigative work, allow us to evaluate these structures on an individual in a three-dimensional way in a 3-year-old human subject. We develop an educational tool on a paediatric dentistry subject in order to evaluate this 3D model. The students gave a very positive evaluation of this tool, and in future research, this 3D model could help teachers and students in the field of dentistry.

## Figures and Tables

**Table 1:** Free response to the question: Do you have any suggestions regarding this type of practice?

Free Comments	N Students
No suggestions	7
Carry out the same type of exercise but with wrist radiographies	4
See more clinical cases of a different type	6
Teach how to properly use and interpret this type of method	2
View slices of tooth buds	1
The relief of the body of the mandible should be stronger/Include images of “normal” anatomy (with no colour)	2
Teach how to use software that the faculty uses for seeing the CT scans. Observe how to control a scanner and make slices	2
Identify more anatomic structures such as vessels, nerves and muscular insertions not only in hard tissues (but with magnetic resonance)	1
Further training with x-rays as there is a lack of knowledge	2
Very useful practice for recognizing the anatomic structures and for the correct diagnosis of each case	2
See complete scanner in various planes and slices	3

## Data Availability

Data is available upon reasonable request to the correspondence author.
